# Bacterial Isolation from Natural Grassland on Nitrogen-Free Agar Yields Many Strains Without Nitrogenase

**DOI:** 10.3390/microorganisms13010096

**Published:** 2025-01-06

**Authors:** Amrit Koirala, Nabilah Ali Alshibli, Bikram K. Das, Volker S. Brözel

**Affiliations:** 1 Department of Biology and Microbiology, South Dakota State University, Brookings, SD 57007, USA; amrit.koirala@jacks.sdstate.edu (A.K.); nabilah.alshibli@jacks.sdstate.edu (N.A.A.); bikram.das@jacks.sdstate.edu (B.K.D.); 2 Department of Biochemistry, Genetics and Microbiology, Forestry and Agricultural Biotechnology Institute (FABI), University of Pretoria, Pretoria 0028, South Africa

**Keywords:** culturable, diazotroph, nitrogenase, nitrogen-fixing, prairie, soil

## Abstract

Nitrogen inputs for sustainable crop production for a growing population require the enhancement of biological nitrogen fixation. Efforts to increase biological nitrogen fixation include bioprospecting for more effective nitrogen-fixing bacteria. As bacterial nitrogenases are extremely sensitive to oxygen, most primary isolation methods rely on the use of semisolid agar or broth to limit oxygen exposure. Without physical separation, only the most competitive strains are obtained. The distance between strains provided by plating on solid media in reduced oxygen environments has been found to increase the diversity of culturable potential diazotrophic bacteria. To obtain diverse nitrogen-fixing isolates from natural grasslands, we plated soil suspensions from 27 samples onto solid nitrogen-free agar and incubated them under atmospheric and oxygen-reducing conditions. Putative nitrogen fixers were confirmed by subculturing in liquid nitrogen-free media and PCR amplification of the *nifH* genes. Streaking of the 432 isolates on nitrogen-rich R2A revealed many cocultures. In most cases, only one community member then grew on NFA, indicating the coexistence of nonfixers in coculture with fixers when growing under nitrogen-limited conditions. To exclude isolates able to scavenge residual nitrogen, such as that from vitamins, we used a stringent nitrogen-free medium containing only 6.42 μmol/L total nitrogen and recultured them in a nitrogen-depleted atmosphere. Surprisingly, PCR amplification of *nifH* using various primer pairs yielded amplicons from only 17% of the 442 isolates. The majority of the *nifH* PCR-negative isolates were *Bacillus* and *Streptomyces*. It is unclear whether these isolates have highly effective uptake systems or nitrogen reduction systems that are not closely aligned with known nitrogenase families. We advise caution in determining the nitrogen fixation ability of plants from growth on nitrogen-free media, even where the total nitrogen is very limited.

## 1. Introduction

Synthetic nitrogen fixation by the Haber–Bosch process revolutionized agriculture, but the cost and negative impacts thereof on the environment are driving the quest for the enhancement of biological nitrogen fixation (BNF) [1,2]. BNF is carried out by a diverse group of prokaryotes from Archaea and Bacteria, termed diazotrophs, and is currently the second largest contributor of fixed nitrogen to the biosphere [3]. It is estimated to contribute 128 Tg N year^−1^ in terrestrial ecosystems and 140 Tg N year^−1^ in marine ecosystems [4]. Nitrogen-fixing bacteria reduce atmospheric nitrogen via the nitrogenase enzyme complex and are divided into two major types: (1) symbiotic nitrogen-fixing bacteria, which form a symbiotic relationship with legumes such as *Rhizobium*, with actinorhizal plants such as *Frankia* and *Cyanobacteria* associated with cycads, and (2) free-living nitrogen fixers belonging to genera such as *Azotobacter* [5]. Most marine diazotrophs [6], terrestrial cyanobacteria [7], methanogenic Archaea [8], and Firmicutes [9] can fix nitrogen on their own. Efforts are underway to find both symbiotic and free-living strains able to contribute more nitrogen to plant growth.

Nitrogenase is a complex heteroenzyme composed of two components. Component I, also called nitrogenase reductase or Fe-protein, is a homodimer of NifH and is encoded by *nifH*. The second component is dinitrogenase, which is a heterotetramer consisting of two proteins. The alpha chain is encoded by *nifD*, and the beta chain is encoded by *nifK* [10]. Among these three structural components, *nifH* genes have been used extensively for the study of diazotroph diversity [11], and multiple universal and group-specific primers have been developed for the study of the *nifH* gene [12]. Phylogeny based on the *nifH* gene classifies *nifH* and its homologs into clusters, but the increase in sequence availability has driven increasingly fine-tuned classification [13]. Cluster I consisted of NifH and VnfH from aerobic and facultative anaerobic proteobacteria and cyanobacteria. Cluster II consisted of VnfH and NifH from some archaea. Cluster III consists of NifH from strict anaerobes, and Cluster IV includes all NifH homologs, such as NflH and Bch/ChlL [11]. Although the nitrogenase enzyme complex is the only enzyme system in prokaryotes known to fix nitrogen, some studies have indicated the existence of alternative enzymes that can reduce the dinitrogen present in the atmosphere into a combined form, such as ammonia. Earlier reports of an oxygen-tolerant nitrogenase in *Streptomyces thermautotrophicus* [14] were recently disproved [15]. In 2020, Higdon et al. used genomics and a machine learning model to identify putative BNF components even in the absence of *nif* genes [16]. Whether all BNF occurs through the recognized nitrogenase complex is currently unresolved.

Nitrogenase is irreversibly inactivated by O_2_, but many diazotrophs are aerobes that use oxygen as a terminal electron acceptor. Symbiotic diazotrophs are protected in legume nodules by plant-produced leghemoglobin, which binds oxygen with high affinity [17]. Aerobic free-living diazotrophs need additional cellular resources to decrease the redox potential of nitrogenase. Hence, free-living diazotrophs are less efficient and fix approximately one-tenth of the total atmospheric N_2_ fixed by symbiotic diazotrophs [18]. However, given the omnipresence of these bacteria in diverse habitats, nitrogen fixation by free-living diazotrophs has been predicted to contribute a significant amount of fixed nitrogen to biological nitrogen fixation [3]. Some diazotrophs belonging to the genera *Paenibacillus*, *Herbaspirillium*, *Klebsiella*, and *Azospirillum* occur in close association with plants in the rhizosphere. They are termed associative diazotrophs and depend on plant exudates for carbon sources [19]. For the purpose of this research, free-living nitrogen-fixing bacteria also include associative diazotrophs, as it is very difficult to distinguish between associative and free-living bacteria in a soil environment heavily occupied by sod-forming grasses that have dense entangled root systems.

Nitrogen-fixing bacteria have been isolated using several culture-based methods [20,21,22]. As nitrogenase is extremely sensitive to oxygen, most of these methods rely on the use of semisolid agar or broth to limit oxygen exposure. More recently, the use of solid media in reduced oxygen environments has been found to increase the diversity of culturable potential diazotrophic bacteria [21]. Confirming the nitrogen-fixing nature of isolates has been pursued using genetic (*nifH*) and biochemical (nitrogenase activity) approaches. Nitrogenase activity is quantified either by the acetylene reduction assay, which requires GC-MS [23] or by the incorporation of ^15^N, which requires ^15^N_2_ gas incubation followed by isotopic mass spectrometry. As both approaches require specialized, expensive equipment, screening of putative isolates typically involves PCR amplification of the most conserved nitrogenase gene, *nifH* [12]. All the *nifH* primers described to date are degenerate, and none of the universal primers developed have 100% coverage of the diazotrophic community [24]. In addition, *nifH* primers are prone to amplifying homologous genes that do not have any role in nitrogen fixation. Therefore, a culture-independent method using *nifH* as a marker gene is prone to bias caused by primary degeneracy and low coverage. In addition, culture-independent studies failed to provide the bacterial cultures that are required for further characterization of diazotrophy.

Prairies are vast extents of arid/semiarid grassland, occupying the vast majority of the central United States [25]. Biological soil crusts (BSCs) in these grasslands are unique ecosystems where diverse microorganisms live in close proximity to grass roots and dead and decaying organic matter [26]. BSCs are the major source of nitrogen in arid and semiarid lands [27]. In areas with minimal human activity, microorganisms present in BSCs play a vital role in the fixation of carbon and nitrogen [28]. Free-living diazotrophic bacteria contribute to a substantial amount of nitrogen in grassland ecosystems where symbiotic nitrogen fixation is rare [29]. A study of asymbiotic nitrogen-fixing microorganisms in natural, sown, and partially degraded alpine grasslands on the Tibetan Plateau identified several diazotrophic bacteria from the phylum Proteobacteria and revealed significant differences in the diversity of asymbiotic nitrogen-fixing bacteria in these grasslands [30]. Similarly, a study of nitrogen-fixing bacteria associated with Switchgrass in the native tallgrass prairie of northern Oklahoma reported several species of *Alphaproteobacteria* and *Bacillota* with *nifH* genes. Although other studies [31,32] have also shown that nitrogen fixation in soil is significantly affected by the grass species abundant in a given grassland; the physiology and limitations of free-living diazotrophs in natural grassland ecosystems are virtually unexplored, indicating the need for further study [3]. There remains a dire need to obtain free-living nitrogen-fixing bacteria that can contribute combined nitrogen to grass-derived crops such as corn, wheat, sorghum, and rice.

The purpose of this study was to obtain diazotrophic isolates from natural grasslands and explore the diversity of free-living nitrogen-fixing bacteria in this ecosystem. The objectives were to (1) isolate diazotrophs on nitrogen-free agar under both atmospheric and reduced oxygen conditions; (2) confirm putative nitrogen fixers by subculturing in liquid nitrogen-free medium in an ammonia-depleted atmosphere; and (3) PCR amplify and sequence the 16SrRNA and *nifH* genes.

## 2. Materials and Methods

### 2.1. Source of Isolates

Soil samples were collected from Sioux Prairie, a natural grassland preserved and maintained by The Nature Conservancy, located at 231st Street, Colman, SD 57017, USA (44°2′8″ N 96°46′0″ W), at 524 m elevation. This site has a typical upper mid-west American continental climate with four distinct seasons, where winters are cold and dry, and summers are warm and semihumid. The average annual precipitation is 635 mm, the highest temperature in summer is 32 °C, and the average temperature in winter is −12 °C. Samples were collected from three transects located 5 m apart, with 9 samples collected from each transect every 1 m, resulting in a total of 27 samples. The soil cores were collected 10 cm below the surface and were 2.5 cm in diameter. All the soil samples were collected into 50 mL conical tubes and stored on ice until processing in the laboratory.

### 2.2. Isolation of Potential Diazotrophic Bacteria

Potential diazotrophic bacteria were isolated using nitrogen-free medium (NFM), which supports the growth of diverse nitrogen-fixing bacteria [33]. For this study, we added arabinose, mannitol and malic acid as additional carbon sources. For initial isolation, nitrogen-free agar (NFA) was prepared by solidifying the NFM with 15 g/L Noble Agar (Difco^TM^ 214230, Pinellas Park, FL, USA). NFM was composed of glucose (2 g/L), arabinose (2 g/L), mannitol (2 g/L), malic acid (2 g/L), K_2_HPO_4_ (0.2 g/L), KH_2_PO_4_ (0.5 g/L), MgSO_4_·7H_2_O (0.2 g/L), FeSO_4_·7H_2_O (0.1 g/L), Na_2_MoO_4_·2H_2_O (0.005 g/L), and NaCl (0.2 g/L). The pH of the NFMs was set to 7.2. Sugars and organic acids were adjusted to pH 7.2 and autoclaved individually before they were added to the sterile base medium. The nitrogen-free medium and its major constituents were tested for organic and inorganic nitrogen compounds (NO_2_^−^, NO_3_^−^, NH_4_^−^, and total nitrogen) at the CAChE Nutrient Analysis Core Facility at Florida International University, Miami, FL, USA.

Soil samples (5 g) were suspended in 45 mL of sterile deionized water in sterile 250 mL Erlenmeyer flasks and mixed by shaking on a shaker for 8 h at room temperature, and serial dilutions were plated on NFA in replicates of six. The inoculated plates were incubated at 28 °C for 15 d under reduced oxygen conditions (n = 3) or ambient oxygen conditions (n = 3). Reduced oxygen conditions were provided by incubating in an airtight chamber with a Gaspak system (BD GasPak^TM^ EZ, 260680, Franklin Lakes, NJ, USA). After 15 d of incubation, the colonies were counted, and 8 isolates were selected from each sample from each incubation condition based on their distinct colony morphology. This process yielded 432 isolates for further processing. The culturable count data were analyzed using Duncan’s new multiple range test.

### 2.3. Purification of Isolates to Obtain Pure Cultures of Potential Diazotrophs

**Figure 1:**
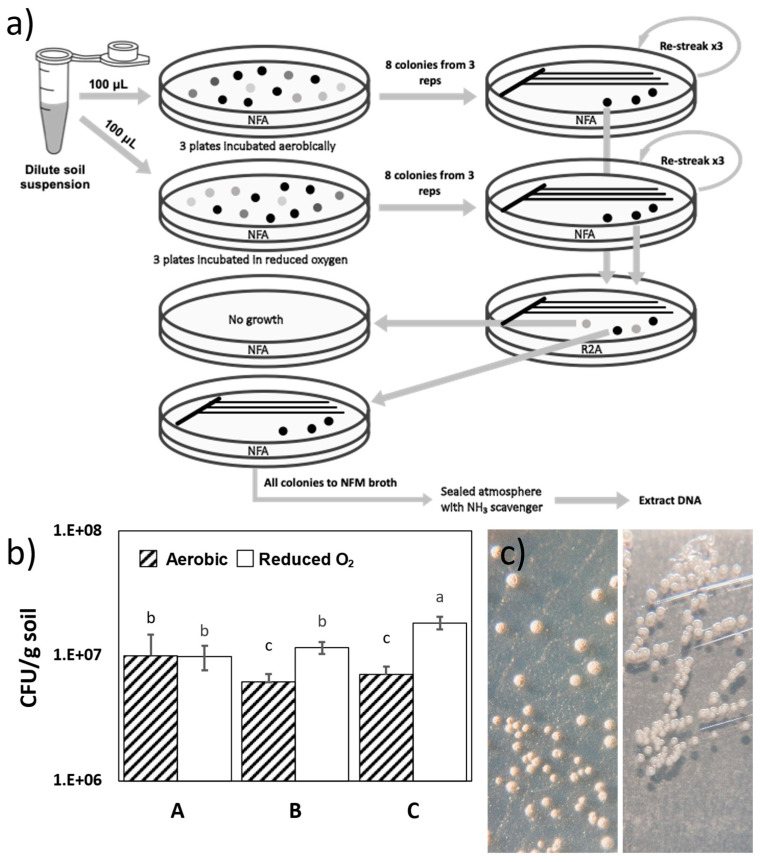
Diversity of isolates obtained on NFA incubated aerobically or under reduced oxygen conditions; (**a**) flow chart illustrating the isolation and processing of putative nitrogen-fixing bacteria from Sioux Prairie soil samples; (**b**) culturable counts on NFA incubated under aerobic and reduced oxygen conditions at 28 °C for 14 d, with letters indicating significant differences as determined by Duncan’s new multiple range test; and (**c**) colony characteristics of an isolate on R2A (left) and NFA (right).

The isolates were subjected to a rigorous protocol to obtain single cultures and to confirm their growth both on NFA and in NFM (Figure 1a). The isolates were streaked on NFA three times to eliminate the possibility of any carryover of combined nitrogen from the original sample supporting the growth of the isolates. The isolates were then subcultured on nitrogen-rich R2A (Difco^TM^ Catalog No. BD218263) and incubated at 28 °C for one week under both aerobic and reduced oxygen conditions. In cases where R2A yielded cocultures of two or more colony morphotypes, each distinct colony was restreaked onto NFA to confirm its ability to grow on NFA by itself. Several such isolates failed to grow on NFA and were discarded as nondiazotrophic cocultures or hitchhikers, while others yielding more than one colony morphotype on R2A both grew on NFA. In some cases, fungi grew out, and these isolates were subcultured on R2A supplemented with cycloheximide (200 mg/mL) and nystatin (40 mg/mL) to suppress fungal growth. The absence of fungus after subculturing was later confirmed by PCR amplification of the eukaryotic internal transcribed spacer region (ITS) using primers ITS5 (5′-GGAAGTAAAAGTCGTAACAAGG-3′) and ITS4 (5′-TCCTCCGCTTATTGATATGC-3′) [34].

Isolates that continued to grow on NFA were subcultured in NF broth and incubated in an airtight chamber with ammonia-binding clinoptilolite (Zeolite Clinoptilolite, Heiltropfen Lab, London, UK). Clinoptilolite has a high binding affinity for ammonia so removes any atmospheric combined nitrogen from the closed space [35]. Isolates able to grow by scavenging NH_3_ from the atmosphere would, therefore, not grow in the chamber containing clinoptilolite. The isolates satisfying all these criteria were processed further, and subcultures were stored in 50% glycerol at −80 °C.

### 2.4. Amplification of the 16S rRNA Sequences and Classification of the Isolates

For genomic DNA, isolates were cultured in R2 broth for 48 h at 28 °C, and DNA was extracted using a DNeasy UltraClean Microbial Kit (Qiagen 12224-50, San Diego, CA, USA). All steps involving DNA and PCR were performed in a PCR hood using filter tips to avoid possible contamination by foreign DNA. The obtained DNA was quantified spectrophotometrically with a NanoDrop spectrophotometer. DNA integrity and quality were assessed electrophoretically by visualizing total genomic DNA resolved on a 0.8% agarose gel, and the DNA was stored at −20 °C. The hypervariable V1 to V3 region of the 16S rRNA gene was amplified using the universal PCR primers 27F (AGA GTT TGA TCM TGG CTC AG) [36] and 518R (GTA TTA CCG CGG CTG CTG G) [37]. PCR was performed in 30 µL reactions consisting of 0.2 µL (5000 U/mL) of Taq polymerase (New England BioLabs, Ipswich, M, USA), 3 µL (10X) of PCR Buffer, 0.6 µL of dNTPs (each 10 mM), 0.6 µL (10 mM) of each primer, and 2.4 µL (25 mM) of MgCl_2_. The PCR conditions were as follows: initial denaturation at 95 °C for 4 min; 30 cycles at 95 °C for 30 s, 50 °C for 45 s, and 72 °C for 1 min; and a final elongation at 72 °C for 10 min. The integrity and quality of the PCR products were checked electrophoretically using a 1% agarose gel and sequenced using Sanger sequencing (GenScript Sequencing Company, Piscataway, NJ, USA). The sequences were aligned and classified using the Sina Sequence Aligner against the SILVA database (https://www.arb-silva.de/aligner/). The aligned reads with references were used to construct a maximum likelihood tree via PhyML via Bayesian-like transformation of aLRT (aBayes) bootstrapping [38]. The phylogenetic tree was visualized and annotated using iTOL [39].

### 2.5. Amplification of nifH Sequences

The amplification of *nifH* was performed using multiple primer pairs under different reaction conditions (Table 1). PCR was performed in 30 μL reactions comprising 0.2 μL (5000 U/mL) of Taq polymerase (New England BioLabs), 3 μL (10X) of PCR Buffer, 0.9 μL of dNTPs (each at a concentration of 10 mM), 0.9 μL (10 mM) of each primer, and 2.5 μL (25 mM) of MgCl_2_. The PCR conditions for the amplification of PolF/PolR were initial denaturation at 94 °C for 5 min, followed by 30 cycles of denaturation at 94 °C for 1 min, annealing at 55 °C for 1 min, and elongation at 72 °C for 30 s, with a final elongation at 72 °C for 5 min. The size of the PolF/PolR PCR product was ~360 bp. To optimize the PCR conditions, nonspecific binders such as BSA (New England BioLabs), 5% DMSO, and 5% glycerol were tested. For the IGK3/DVV and Ueda19F/Ueda407R primer pairs, the annealing temperatures were 59 °C and 52 °C, respectively. Genomic DNA from *Herbaspirillum seropedicae* ATCC 35,892 and *Bradyrhizobium diazoefficiens* USDA 110 was used as a positive control, and *Escherichia coli* MG1655 was used as a negative control. The product size obtained using these primers ranges from ~360 to 400 bp.

## 3. Results

### 3.1. Isolation of Putative Diazotrophs

Bacterial isolates with diverse morphological features were obtained from all 27 samples under both incubation conditions. The culturable count was highest in transect C under reduced oxygen conditions (Figure 1b). Two of the three transects yielded significantly more isolates under reduced oxygen incubation conditions, as determined using Duncan’s new multiple-range test. The isolates generally produced colonies less than 1 mm in diameter and were mucoid or dry and cream- or white-colored. After subsequent incubation for more than 14 d, many of them produced a wrinkled surface, indicating the formation of spores (Figure 1c).

Eight isolates were sampled from the highest dilutions showing growth from both aerobic and reduced oxygen incubations of each of the 27 samples, yielding 432 isolates. These isolates were further processed to obtain single cultures, as outlined in Figure 1a. This required repeated subculturing on NFA and a transition between nitrogen-rich R2A and nitrogen-deficient NFA. When subcultured on R2A, most of the isolates produced distinct colony morphologies, but in many cases, two or even three colony morphotypes were observed. All the isolates yielding more than one morphotype on R2A were confirmed for growth on NFA, and in most cases, only one of these isolates grew on NFA, indicating the coexistence of nondiazotrophic bacteria in coculture with diazotrophic bacteria when growing under nitrogen-limited conditions on NFA. Bacteria unable to grow on NFA were viewed as hitchhikers and were not pursued further. Some of the isolates yielded fungi together with bacteria on R2A, and these bacteria were eliminated by the addition of the fungal suppressants cycloheximide and nystatin. The isolates satisfying our criteria for single cultures able to grow on NFA were then evaluated for their ability to grow in liquid NFM without agar and in a nitrogen-depleted atmosphere. This rigorous process of purification yielded 471 putative diazotrophic bacteria in a single culture. This number exceeded 432 because in some cases, initial colonies on NFA were found to comprise more than one strain able to grow on NFA.

### 3.2. Diversity of Isolates from NFA

**Figure 2:**
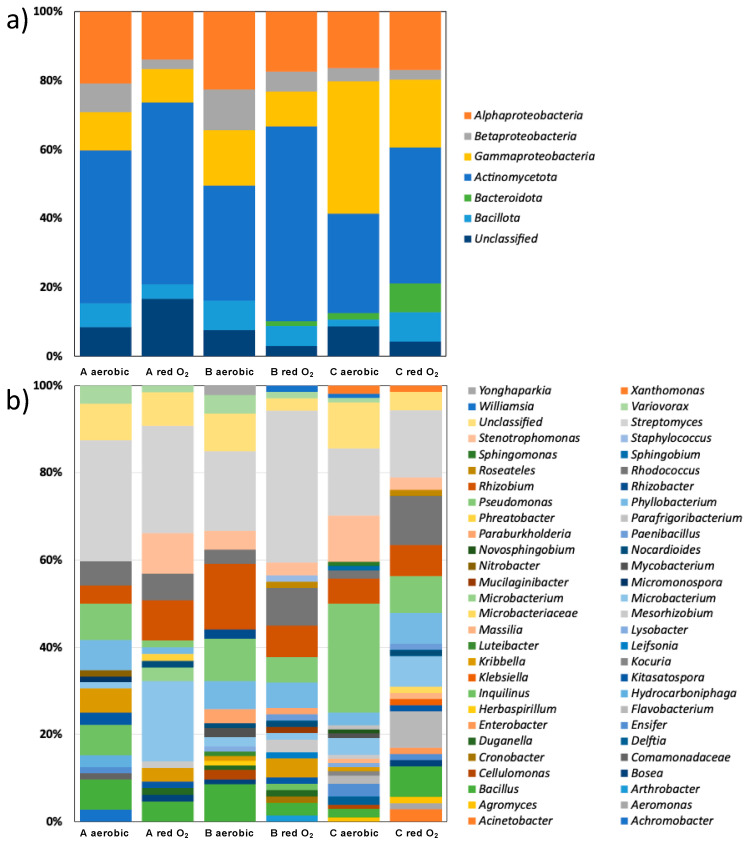
Diversity of purified isolates determined by the V1–V3 region of 16S rRNA sequences; (**a**) shown at the phylum/class level, and (**b**) at the genus level.

The V1-V3 16S rRNA gene sequences were evaluated for sequence quality using RDPII, which yielded 471 partial 16S rRNA sequences. These 471 sequences were classified using the SILVA database with an 80% cutoff parameter. Of these, 93% (442) were classified as bacteria, and the remainder had a similarity percentage of less than 80%, so they were labeled unclassified. All 442 classified isolates belonged to one of four phyla: *Actinomycetota* (previously *Actinobacteria*) (198, 42%), *Pseudomonadota* (previously *Proteobacteria*) (207, 43%), *Bacillota* (previously *Firmicutes*) (28, 6%), and *Bacteroidota* (previously *Bacteroidetes*) (9, 2%). The *Pseudomonas* isolates included *Alphaproteobacteria* (87, 18%), *Gammaproteobacteria* (91, 19%), and *Betaproteobacteria* (29, 6%) (Figure 2a). The isolates were distributed across three different transects with some variation. *Actinomycetota* were dominant in transect A, but *Gammaproteobacteria* were more abundant in transect C. Additionally, *Bacteroidota* were absent in transect A. Of the 58 genera detected, *Bacillus*, *Bosea*, *Kitasatospora*, *Kribbella*, *Mesorhizobium*, *Microbacterium*, *Nocardioides*, *Phyllobacterium*, *Pseudomonas*, *Rhizobium*, *Rhodococcus*, *Stenotrophomonas*, *Streptomyces*, and *Variovorax* were present in all three transects (Figure 2b). Of the 58 genera, 24 grew under both aerobic and oxygen-limiting conditions, the most abundant of which were *Streptomyces*, *Pseudomonas*, *Rhizobium*, *Rhodococcus*, *Stenotrophomonas*, *Phyllobacterium*, *Microbacterium*, and *Bacillus* (Figure 2b). Nineteen genera were obtained under aerobic incubation conditions only, and fourteen genera were unique to reduced oxygen conditions (Table 2).

**Figure 3:**
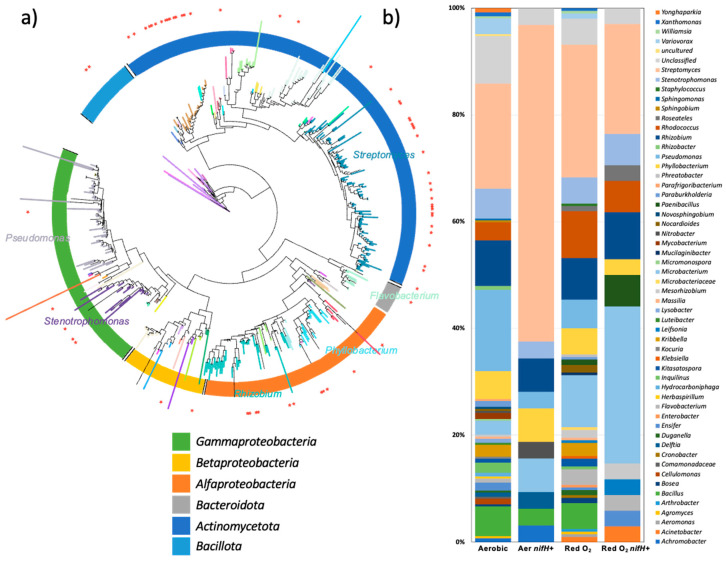
(**a**) Molecular phylogenetic analysis of partial 16S rRNA sequences (V1–V3) obtained from potential diazotrophic isolates via maximum likelihood in PhyML via the aBayes analysis method. Branch tips are colored according to the genus classification, and unclassified sequences are not presented. The red stars on the outer ring indicate isolates that yielded *nifH* sequences by PCR. (**b**) Distribution of potential diazotrophs compared to *nifH*-positive isolates, shown by incubation conditions.

The phylogenetic tree of the isolates (Figure 3a) shows the potential diazotrophs distributed according to their phyla and classes. The most abundant phylum was *Pseudomonadota*, which branched out into the *Alphaproteobacteria*, *Betaproteobacteria*, and *Gammaproteobacteria* clusters. *Rhizobium* and *Phyllobacterium* were the most abundant *Alphaproteobacteria*. The isolates from the family *Burkholderiales* dominated the class Betaproteobacteria. *Variovorax* was the most abundant *Betaproteobacteria*. *Pseudomonas* and *Stenotrophomonas* were the most abundant *Gammaproteobacteria*. Another Gram-negative phylum isolated in this study was *Bacteroidota*, which was dominated by *Flavobacterium.* Both the Gram-positive phyla *Actinomycetota* and *Bacillota* were detected. *Actinomycetota* was the most dominant, with four clusters represented by *Streptomyces*, *Rhodococcus*, *Kribbella*, and *Microbacterium*. *Bacillota* were mainly represented by *Bacillus*, and very few were represented by *Paenibacillus* or *Staphylococcus*.

### 3.3. Survey of nifH Among the Isolates

Genomic DNA from all the isolates was evaluated for the presence of *nifH* using four different primer sets (Table 1). The PolF/PolR primer pair yielded amplicons from 75 (17%) of the 442 isolates classified as bacteria by RDP (Table 2). Neither the UEDA19f/UEDA407r, IGK3/DVV nor 19f/NifH3 primer pair yielded amplicons from any of these isolates, while they amplified *nifH* from the positive control strains *H. seropedicae* and *B. diazoefficiens*. The majority of *nifH* sequences were obtained from isolates identified by 16S rRNA phylogeny as *Streptomyces* (30), *Microbacterium* (12), *Rhizobium* (5), *Rhodococcus* (4), *Stenotrophomonas* (4), *Paenibacillus* (4), and *Nocardioides* (4) (Figure 3b, Table 2).

### 3.4. Chemical Analysis of NFM

The growth of bacteria devoid of *nifH* on NFA prompted us to measure the amount of nitrogen in the NF medium and its key components. We intentionally selected an isolation medium free of vitamins and cofactors because several of these contain nitrogen. The NFA contained trace quantities of nitrogen (Table 3). Nitrate, ammonium, and total N were below the detection limits for the two organic components of NFMs, noble agar and arabinose, but the purest source of water we had access to contained 2.57 μmol/L of total nitrogen. The 6.42 μmol/L of total nitrogen in our NFMs may be due to the presence of trace quantities of the various medium constituents, which were all of analar grade. *E. coli* MG1655 and *B. subtilis* 158 streaked onto NFA did not grow to form colonies visible to the naked eye, even after extended incubation, showing that our NFA did not contain sufficient N to support the detectable growth of heterotrophic bacteria.

To determine the approximate number of cells that can be formed using all 6.42 μmol/L, we used the nitrogen content reported for growth under nutrient-limiting conditions. The following calculations assume that the cells have taken up all the N in the medium by highly efficient scavenging, displaying highly effective uptake systems and that no N would remain in the medium. Under N limitation, marine bacterioplankton cells contain approximately 20 fg N per cell [42], or 1.428 fmol. Therefore, 6.42 μmol N (6.42 × 10^9^ fmol) supported the growth of 4.5 × 10^9^ (6.42 × 10^9^/1.428) cells/L or 4.5 × 10^6^ cells/mL. Similarly, under N-limiting conditions, *E. coli* contains 6.5% N per dry mass [43,44]. Assuming that one *E. coli* cell is 1 pg wet weight and that the dry mass of bacteria is 30%, 0.33 pg of dry matter is included. One such cell, therefore, contains ((6.5/100) × 0.33 pg) 2.1 × 10^−2^ pg or 21 fg of nitrogen, or 1.5 fmol. This would be sufficient to form (6.42 × 10^9^/1.5) cells per liter or 4.28 × 10^6^ cells per mL. At small cell sizes, a liquid culture will not show turbidity at this cell density. Collectively, these results indicate that there was insufficient N in our NFA to support the growth of single cells to form colonies visible to the eye.

## 4. Discussion

### 4.1. Comparisons to Prior Studies

The grassland of Sioux Prairie yielded diverse bacteria that were able to grow under nitrogen-free conditions. The isolation and enumeration of nitrogen-fixing bacteria have been performed mostly using soft agar in tubes to facilitate a reduced oxygen environment [45], but we followed the approach of Mirza and Rodrigues [21] by plating directly onto nitrogen-free agar and incubating in a reduced oxygen atmosphere. This method yielded, on average, 10^7^ CFU/g of potential free-living diazotrophs, which is more than 10^4^ times the estimated 10^3^ CFU/gm reported for the wheat rhizosphere using semisolid NFB [46]. Other isolation projects that used semisolid media also reported lower counts of putative nitrogen-fixing bacteria, 10^4^ CFU/gm in the chickpea rhizosphere [47] and 10^4^–10^5^ CFU/gm in the sugarcane rhizosphere [48]. The vastly higher isolation number is likely due to the spatial separation of slow-growing strains from other fast-growing strains on the surface of the agar, thereby allowing enough time for slow growers to form visible colonies on agar plates without being outcompeted by adjacent faster growers [21]. Furthermore, culture-independent quantification of *nifH* in summer perennial grasses by qPCR revealed a copy number of 1–5 × 10^6^ per gram [49], which is closer to our number of 10^7^/gm. This indicates that the isolation approach used here is far superior to the use of soft agar.

The isolates obtained on NFA represented a diverse bacteriota across 58 genera, belonging to Pseudomonadota (43%), Actinomycetota (42%), Bacillota (6%), and Bacteroidota (2%). Nitrogen-fixing isolates from the Andean wheat rhizosphere also belong to these four phyla [46]. Similarly, isolates from Amazonian forest soil also represented these four phyla [21]. A comparison of the diversity obtained using solid agar (incubated under atmospheric and reduced oxygen atmospheres) versus semisolid media showed that plating on solid agar under reduced oxygen conditions yielded the greatest diversity [21]. We did not observe a significant difference between the CFU count under aerobic and reduced oxygen conditions. Additionally, most of the bacteria obtained only under reduced oxygen incubation by Mirza and Rodriguez were also obtained under aerobic incubation conditions in our study. Slower-growing taxa and taxa susceptible to antagonistic action of others were able to grow on solid agar due to spatial separation, which was not supported by soft agar. While incubation under reduced oxygen allowed for the isolation of multiple genera not obtained in regular air, several taxa were obtained only under atmospheric oxygen. The use of more than one incubation condition, therefore, expanded the diversity of the isolates obtained.

### 4.2. Isolates with Uncertain Nitrogen-Fixing Ability

Many of the isolates obtained in this study are well-known diazotrophs (Table 2), members of *Alfa-*, *Beta-*, and *Gamma-Proteobacteria*, *Bacillota*, and *Bacteroidota*, but one-quarter of the isolates were *Streptomyces*, a member of the *Actinobacteriota*. A culture-independent study of nitrogen-fixing bacteria associated with Switchgrass [50] using the *nifH* 3 and *nifH* 4 primers revealed diazotrophic species from *Alpha-*, *Beta-*, *Delta-*, *Gamma-Proteobacteria*, and *Bacillota* only. A study of Tibetan grassland soils using PolF/R primers reported *Cyanobacteriota*, *Pseudomonadota*, and *Verrucomicrobiota* [51]. The absence of diazotrophic *Actinobacteriota nifH* in culture-independent studies could be the result of *nif* primer bias. Conversely, the large number of *Actinobacteria* we obtained may be due to the inability of our selective medium (NFA) to distinguish between true diazotrophs and oligotrophic bacterial species. There have been several reports of false-positive diazotrophic fungi and bacteria because of their ability to grow in nitrogen-free media [52]. In 1969, Hill and Postgate reported that not all isolates growing on N-free media are able to fix N [53]. To preempt this challenge, we eliminated all N-containing components, such as yeast extract and vitamins, that could be potential sources of nitrogen and performed a chemical analysis of the media for total nitrogen, ammonia, nitrite, and nitrate content. The nitrogen-free media used in this study contained only 6.42, 1.19, 0.31, and 0.97 μmol/L total nitrogen, ammonia, nitrite, and nitrate, respectively. Nitrogen was not detected in the noble agar used to prepare the solid NFA. Additionally, to ensure that no ammonia in the atmosphere supported the growth of bacteria, we incubated the isolates in sealed containers with clinoptilolite, an ammonia scavenger [35]. These strict measures should only encourage true diazotrophs to grow on NFA, but the possibility of very efficient N scavengers growing on our NFA cannot be eliminated completely.

The most abundant isolates in this study were *Streptomyces*, and there is no genomic sequence evidence that these contain the core *nifHDKENB* cluster [13]. However, there are multiple reports of *Streptomyces* strains grown on nitrogen-free media. It has been reported that *S. thermoautotrophicus* fixes nitrogen via an oxygen-insensitive novel nitrogenase that does not reduce acetylene to ethylene [14]. This claim was recently refuted by a multi-institution group that failed to observe nitrogen fixation in *S. thermoautotrophicus* [15]. However, other *Streptomyces* species are still frequently reported to fix nitrogen [54] or to play beneficial roles in plants [55,56]. Recently, an agar-degrading, nitrogen-fixing *S. lavendulae* strain was reported to contain *nifU* [57] but not any of the nitrogenase core genes. The second most abundant *Actinobacteriota* in this study, *Microbacterium*, has also been previously isolated in nitrogen-free media [46] and reported to fix nitrogen [58], but there is still a lack of genomic evidence [13]. Species of *Rhodococcus* are also frequently isolated in nitrogen-free media [59,60], and their ability to scavenge atmospheric NH_3_ has been studied in detail [61]. Similarly, *Mycobacterium* has been reported to fix nitrogen [58], but a novel glucosylglycerate hydrolase enzyme has been suggested to be responsible for the recovery of *M. hassiacum* from nitrogen starvation [62]. Similarly, there have been multiple reports of diazotrophy in other species of *Actinobacteriota*, such as *Agromyces*, *Williamsia*, and *Kocuria*, but genome sequence analysis suggested that *Frankia* and *Propionibacterium* are the only two *Actinobacteriota* with a complete minimal set of nitrogenase genes in the sequenced genome, which is supported by biochemical evidence [13].

*Bacillus* was the most abundant genus of *Bacillota* in this study. While members of the classical genus *Bacillus* are widely reported as nitrogen fixers, the genus has undergone multiple revisions, and the diazotrophy of *Bacillus* needs some clarification. Most *Bacillus* strains found to have *nif* genes, such as *B. polymyxa* and *B. macerans*, have been reclassified as *Paenibacillus*, and diazotrophy among aerobic endospore-forming Firmicutes is restricted to the genus *Paenibacillus* only [9]. The only *Bacillus* strains that possess all six core *nif* genes, *B. caseinilyticus* and *B*. *nealsonii*, are extremophiles and have recently been reclassified as *Evansella caseinilyticus* [54] and *Niallia nealsonii* [54]; therefore, no members of the genus *Bacillus* contain a *nifHDKENB* cluster. This is in contrast to multiple reports of diazotrophic *Bacillus* isolates classified as *B. subtilis*, *B. cereus*, and *B. licheniformis*, supported only by culture evidence for growth on nitrogen-free media [63,64,65]. However, none of these claims have been supported by genetic evidence. We were not able to obtain *nifH* PCR amplicons using any of the commonly used primer sets. Understanding the mechanisms underlying the growth of *Bacillus* on nitrogen-free media is urgently needed.

### 4.3. Research Implications

There is a growing need to replace synthetic fertilizers with biological solutions. In contrast to leguminous crops, the other major crops do not offer nodules to nitrogen-fixing bacteria [66]. Currently, applied free-living nitrogen-fixing bacteria do not provide sufficient nitrogen to replace fertilizer [67], leading to continued bioprospecting for free-living nitrogen-fixing bacteria. The majority of isolates obtained through the stringent isolation and purification protocol did not yield *nifH* amplicons by PCR, using multiple sets of degenerate primers and amplification conditions. The recovery rate of *nifH* sequences from the isolates using the PolF/R primer set was only 17%, which is very low compared to the 79% for *nifH*-positive isolates reported by Mirza and Rodriguez [21]. A lower degree of *nifH* recovery has also been observed in other studies [9,68,69]. This suboptimal performance of *nifH* PCR is partially due to the less conserved nature of the *nifH* gene. The *nifH* gene varies considerably across known diazotrophs and does not have a single fully conserved region among all diazotrophs. This has led to the design of many *nifH* PCR primer sets optimized for specific groups of bacteria [6]. Additionally, all the primers designed thus far are substantially degenerate, decreasing the specificity of the amplification [12] while also yielding non-*nifH* amplicons. These findings indicate that nitrogen fixation cannot be excluded based on *nifH* PCR alone.

Isolation on stringent nitrogen-free isolation and purification media may point to nitrogen fixation. The large number of such isolates whose genomes do not contain genes homologous to known nitrogenases point to a major gap in knowledge. To grow, bacteria require combined nitrogen to synthesize amino acids, nucleic acids, and other compounds, so growth without combined nitrogen is technically not possible. Thus, bacterial growth in culture media devoid of combined nitrogen can only occur if the cells have highly efficient uptake systems that scavenge residual nitrogen or if they use a yet unknown system to reduce atmospheric nitrogen.

## 5. Conclusions

The use of solid nitrogen-free agar yielded diverse culturable diazotrophic bacteria from natural grassland soil. The majority of these isolates also grew in nitrogen-free liquid medium in an ammonia-depleted atmosphere, supporting nitrogen fixation over scavenging of residual atmospheric ammonia. The total nitrogen in the NFM was 6.4 μmol/L, sufficient to yield at most 4.28 × 10^6^ cells per mL and insufficient to be visible to the human eye. Surprisingly, a high proportion of isolates lacked detectable *nifH.* The *nifH*-deficient isolates included members of nitrogen-fixing genera such as *Rhizobium* but also many *Bacillus* and *Streptomyces*, which are not known to contain nitrogenase genes. Whether isolates of genera such as *Bacillus* and *Streptomyces* grow in nitrogen-free media due to highly effective ammonia uptake systems or to a nitrogen reduction system not closely aligned to the nitrogenase family requires further study. We advise caution in determining the nitrogen fixation ability of bacteria from growth on nitrogen-free media, even where the available nitrogen is very limited.

## Figures and Tables

**Table 1:** Primers used to amplify the *nifH* gene from isolates.

Primers		Reference
PolF PolR	TGCGAYCCSAARGCBGACTC ATSGCCATCATYTCRCCGGA	[24]
IGK3 DVV	GCIWTHTAYGGIAARGGIGGIATHGGI ATIGCRAAICCICCRCAIACIACRTC	[12]
Ueda19F Ueda407R	GCIWTYTAYGGIAARGGIGG AAICCRCCRCAIACIACRTC	[40]
19f nifH3 nifH11 nifH22	GCIWTYTAYGGIAARGGIGG ATRTTRTTNGCNGCRTA GAYCCNAARGCNGACTC ADWGCCATCATYTCRCC	[41]

**Table 2:** Genera isolated in this study and detection of *nifH* by PCR with the PolF/PolR primer pair.

Genus	Isolation Conditions	Total Isolates	*nifH* PCR
*Achromobacter*	Aerobic	2	1
*Acinetobacter*	Reduced oxygen	2	1
*Aeromonas*	Reduced oxygen	1	-
*Agromyces*	Both	2	-
*Arthrobacter*	Reduced oxygen	1	-
*Bacillus*	Both	25	1
*Bosea*	Both	3	1
*Cellulomonas*	Aerobic	3	-
*Comamonadaceae*	Aerobic	1	-
*Cronobacter*	Reduced oxygen	1	-
*Delftia*	Aerobic	2	-
*Duganella*	Both	3	-
*Ensifer*	Both	5	-
*Enterobacter*	Reduced oxygen	1	-
*Flavobacterium*	Both	8	1
*Herbaspirillum*	Aerobic	1	-
*Hydrocarboniphaga*	Aerobic	2	-
*Inquilinus*	Both	6	-
*Kitasatospora*	Both	5	-
*Klebsiella*	Reduced oxygen	1	-
*Kocuria*	Aerobic	1	-
*Kribbella*	Both	11	-
*Leifsonia*	Reduced oxygen	1	-
*Luteibacter*	Aerobic	1	-
*Lysobacter*	Aerobic	2	-
*Massilia*	Both	2	-
*Mesorhizobium*	Both	4	2
*Microbacteriaceae*	Reduced oxygen	1	-
*Microbacterium*	Both	27	12
*Micromonospora*	Aerobic	1	1
*Mucilaginibacter*	Reduced oxygen	1	-
*Mycobacterium*	Aerobic	3	1
*Nitrobacter*	Aerobic	1	1
*Nocardioides*	Both	4	2
*Novosphingobium*	Aerobic	1	-
*Paenibacillus*	Reduced oxygen	2	2
*Paraburkholderia*	Both	4	-
*Parafrigoribacterium*	Aerobic	1	-
*Phreatobacter*	Reduced oxygen	1	-
*Phyllobacterium*	Both	24	2
*Pseudomonas*	Both	52	1
*Rhizobacter*	Aerobic	2	-
*Rhizobium*	Both	39	6
*Rhodococcus*	Both	27	4
*Roseateles*	Reduced oxygen	2	1
*Sphingobium*	Aerobic	1	-
*Sphingomonas*	Aerobic	1	-
*Staphylococcus*	Reduced oxygen	1	-
*Stenotrophomonas*	Both	25	4
*Streptomyces*	Both	104	30
*Variovorax*	Both	10	-
*Williamsia*	Both	2	-
*Xanthomonas*	Both	3	1
*Yonghaparkia*	Aerobic	2	-
Unclassified Actinobacteria	Aerobic	2	-
Unclassified *Alphaproteobacteria*	Aerobic	1	-
Total		442	75

**Table 3:** Chemical analysis of NFM, water, arabinose, and noble agar used for NFA and NFM.

Sample	N+N (μmol/L)	NO_3_-N (μmol/L)	NO_2_-N (μmol/L)	NH_4_-N (μmol/L)	TN (μmol/L)
Millipore pure water	0.16	0.16	0.01 *	0.16 **	2.57
Noble agar	ND ***	ND	ND	ND	ND
Arabinose	ND	ND	ND	ND	ND
NFM using Millipore pure water	1.28	0.97	0.31	1.19	6.42

* Result reported is less than the laboratory Method Detection Limit (MDL). ** Results reported are less than the laboratory practical quantitation limit (PQL) and greater than or equal to the MDL. *** Not detected, below the detection limit of the method.

## Data Availability

The sequence data were deposited as a Bioproject at NCBI and are available as accession numbers PQ789289-PQ789620 and PQ821742 to PQ821759.
